# Treatment of malignant gastric outlet obstruction with stents: An evaluation of the reported variables for clinical outcome

**DOI:** 10.1186/1471-230X-9-45

**Published:** 2009-06-17

**Authors:** Lene Larssen, Asle W Medhus, Truls Hauge

**Affiliations:** 1 Department of Gastroenterology, Oslo University Hospital, Ullevaal, Department of Gastroenterology, Kirkeveien 166, N – 0407 Oslo, Norway; 2 University of Oslo, P.B 1078 Blindern, 0316 Oslo, Norway

## Abstract

**Background:**

Malignant gastric outlet obstruction (GOO) is commonly seen in patients with advanced gastric-, pancreatic-, duodenal, hepatobiliary or metastatic malignancies. Ten to 25% of patients with pancreatic cancer will develop duodenal obstruction during the course of the disease. Duodenal stenting with self-expandable metal stents is an alternative treatment to surgical bypass procedures. Our aim was to review the published literature regarding treatment of malignant GOO with stents to reveal whether the information provided is sufficient to evaluate the clinical effects of this treatment

**Methods:**

A literature search from 2000 – 2007 was conducted in Pub Med, Embase, and Cochrane library, combining the following search terms: duodenal stent, malignant duodenal obstruction, gastric outlet obstruction, SEMS, and gastroenteroanastomosis.

All publications presenting data with ≥ 15 patients and only articles written in English were included and a review focusing on the following parameters were conducted: 1) The use of graded scoring systems evaluating clinical success; 2) Assessment of Quality of life (QoL) before and after treatment; 3) Information on stent-patency; 4) The use of objective criteria to evaluate the stent effect.

**Results:**

41 original papers in English were found; no RCT's. 16 out of 41 studies used some sort of graded scoring system. No studies had objectively evaluated QoL before or after stent treatment, using standardized QoL-questionnaires, 32/41 studies reported on stent patency and 9/41 performed an oral contrast examination after stent placement. Objective quantitative tests of gastric emptying had not been performed.

**Conclusion:**

Available reports do not provide sufficient relevant information of the clinical outcome of duodenal stenting. In future studies, these relevant issues should be addressed to allow improved evaluation of the effect of stent treatment.

## Background

Malignant gastric outlet obstruction (GOO) is commonly seen in patients with advanced gastric-, pancreatic-, duodenal, hepatobiliary or metastatic malignancies. Ten to 25% of patients with pancreatic cancer will develop duodenal obstruction during the course of the disease [1,2]. GOO may result in nausea and vomiting, leading to dehydration and cachexia, which severely reduces the patients' Quality of Life (QoL).

Traditionally, a surgical by-pass procedure, usually a gastrojejunoanastomosis (GEA), has been the palliative treatment offered, but up to 31% of the patients do not experience sufficient symptom relief following GEA [1,3]. Furthermore, GEA has a peri-operative morbidity as high as 35% and a mortality rate of about 2% in later studies [1,4-7].

Duodenal stenting with self-expandable metal stents (SEMS) is an alternative treatment to surgical bypass procedures. In several studies, this treatment has been evaluated as safe and efficient with a technical success rate of 90–100%, a clinical success rate of 67–100%, a rate of severe complications about 7% and non-severe complication rate about 20% [2,6-47]. Compared with surgery, the patients treated with stents have fewer serious complications and less need for intensive care unit (ICU) [5] Furthermore, the hospital stay is shorter, which is essential in palliative treatment [5,9,20,32,7].

In palliative cancer treatment, improvement of QoL is a primary goal and needs to be addressed when new treatment strategies and procedures are implemented and evaluated. Relief from obstructive symptoms is the most important parameter for evaluating the clinical effect or treatment outcome following duodenal stenting of GOO, but complications, stent patency and need for re-interventions are also parameters influencing QoL. In the available reports, objective criteria of treatment effects are often missing, which make it difficult to compare results and draw conclusions concerning effects of the treatment offered.

To review the published literature regarding treatment of malignant GOO with stents to reveal whether the information provided is sufficient to evaluate the clinical effects of this treatment, and whether QoL has been assessed.

## Methods

A search for published literature for the time period January 2000 – September 2007 was conducted in Pub Med, Embase, and Cochrane library, combining the following search terms: duodenal stent, malignant duodenal obstruction, gastric outlet obstruction, SEMS, and gastroenteroanastomosis. Reference lists were hand-searched for additional literature. Furthermore, reference lists of review articles and metaanalyses from the relevant time period were used to identify additional literature. Abstracts were not included. Only studies presenting data with ≥ 15 patients and only articles written in English were included in the present review. When studies included identical patients, the most recent study was included.(see additional file 1)

The identified studies were reviewed with regard to the following parameters:

1. The use of a graded scoring systems evaluating clinical success

2. Assessment of QoL before and after treatment

3. Information on stent-patency

Stent patency defined as the time period without need for re-intervention

4. The use of objective criteria to evaluate the stent effect

## Results

When applying the search criteria, 41 original papers and four review articles in English were found (See table 1). The number of patients included in the original papers was 15–213. Of the studies using a combined endoscopic/fluoroscopic method for stent placement ten were prospective and 18 retrospective, corresponding numbers for the studies in which only fluoroscopy was applied were 10 and three, respectively. All prospective and retrospective studies are listed in table 2 and 3 respectively. No randomized controlled trials (RTC's) treating ≥ 15 patients with stents were found.

**Table 1:** Characteristics of studies included in the review (n = 41)

Characteristics	n (% of total)
Prospective studies	20 (49%)
Retrospective studies	21 (51%)
Stent deployed by fluoroscopic guidance	13 (32%)
Stent deployed by combined endoscopic/fluoroscopic guidance	28 (68%)

**Table 2:** Prospective studies

Author	Year	Patients (n)
**Jung (16)**	2000	**19**
**Lopera (11)**	2001	**16**
**Pabon (12)**	2001	**29**
**J.H. Kim (13)**	2001	**29**
**Park (14)**	2001	**24**
**Jung (17)**	2002	**39**
**Lee(21)**	2003	**17**
**Tang (22)**	2003	**21**
**Nassif (23)**	2003	**63**
**Holt (26)**	2004	**28**
**Jeong (27)**	2004	**25**
**Johnsson (5)**	2004	**21**
**Hayashi (47)**	2005	**31**
**Yoon (35)**	2006	**82**
**Espinel (36)**	2006	**24**
**Song (42)**	2007	**20**
**Mutignani(43)**	2007	**64**
**J.H Kim (41)**	2007	**213**
**Lowe(44)**	2007	**87**
**Maetani (45)**	2007	**37**

**Table 3:** Retrospective studies

*Author*	*Year*	*Patients (n)*
**Yim (9)**	2001	**29**
**Razzaq (10)**	2001	**23**
**Aviv (18)**	2002	**15**
**Maetani (15)**	2002	**23**
**Adler (2)**	2002	**36**
**M. Kaw (19)**	2003	**18**
**Mittal (6)**	2003	**16**
**Stawawy (20)**	2003	**24**
**G.H. Kim (24)**	2004	**49**
**Lindsay (25)**	2004	**40**
**Telford (29)**	2004	**176**
**Mosler (30)**	2005	**36**
**Bessoud (32)**	2005	**72**
**Del Piano (31)**	2005	**24**
**Maetani (7)**	2005	**22**
**Maire (33)**	2006	**24**
**Kazi (34)**	2006	**23**
**Kiely (37)**	2007	**30**
**T.O. Kim (40)**	2007	**53**
**J. van Hooft (38)**	2007	**62**
**Jeurnink (8)**	2007	**53**

### Clinical effect and scoring systems

To evaluate the clinical effects of stent treatment, 16 out of 41 studies used some sort of graded scoring system (see table 4). The level of oral intake before and after stent treatment was divided into four to five levels, which allows some comparison of the results. The scoring systems used are adapted from studies on dysphagia in esophageal cancer. One of the most frequently used is **G**astric **O**utlet **O**bstruction **S**coring **S**ystem (GOOSS) presented by Adler in 2002 [2] (0 = no/inadequate oral intake, 1 = liquids/thickened liquids, 2 = semisolids/low residue diet, 3 = unmodified diet). This system assigns a point score based on the level of oral intake. Song et al [48] introduced another similar scoring system (0 = able to eat normal diet, 1 = able to tolerate fragmented solid food without vomiting, 2 = able to tolerate soft food without vomiting, 3 = able to tolerate only liquid diet without vomiting, 4 = not able to tolerate any oral intake without vomiting, 5 = vomiting even without oral intake), mostly used in radiological literature, in which vomiting as an important symptom of obstruction is included. The GOOSS score was applied by 6/41 studies, 1/41 applied the Song score and 8/41 used similar graded scores. Furthermore, in 2007 Lowe et al introduced a Gut function score (0 = profuse vomiting or gut not functioning, 1 = nausea and occasional vomiting, 2 = nausea only, 3 = normal gut function). This function score is used in addition to GOOSS and grades the level of nausea and vomiting. At present, the Gut Function Score has only been applied in the study, in which it was originally presented [44].

**Table 4:** Evaluation criteria applied in the reviewed studies (n = 41)

Evaluation criteria	n (% of total)
Quality of Life assessment	0
Objective criteria for stent function	9 (22%)
Clinical effect by graded scoring	15 (37%)
Stent patency	33 (80%)

### QoL in the evaluation of clinical success

No studies had objectively evaluated QoL before or after stent treatment, using standardized QoL-forms (see table 4). Seven of 41 studies used the Karnofsky performance scale before and after stent treatment (A physical performance scale from 100-0, where a scoring of 100 is normal function and 0 is dead).

### Stent patency

Concerning stent patency, 32/41 studies reported on this variable (see table 4), either by reporting the exact number of stent failures and time to failure after stent deployment or by calculating the patency. The rate of re-obstruction was reported in 36/41 studies, the migration rate in 34/41 studies.

### Objective criteria for stent function

An oral contrast examination was performed after stent placement in 9/41 studies (see table 4). Objective quantitative tests of gastric emptying before and after treatment were not performed in any of the evaluated studies.

## Discussion

The present review demonstrates that a graded scoring system for symptom assessment was used in 40% of the evaluated papers. No studies provided information on QoL, although 17% of the studies used the Karnofsky scale. Information on stent patency was given in 80% of the studies and 22% had performed oral contrast examination following stent placement to objectify the stent effect. No studies quantified the effect of stent placement on rate of gastric emptying.

The main complaints of patients suffering from malignant duodenal obstruction are often nausea, severe vomiting, bloating and abdominal pain. It is questionable whether the applied scoring systems in the papers reviewed provide adequate and sufficient information about relief from these symptoms after stent placement. Improvement of symptoms estimated by a dysphagia score provides limited information concerning the effect of duodenal stenting, and should thus be used in combination with a scoring system providing information about the more characteristic symptoms of GOO. The Gut Function Score may be a step in the right direction [44], but this scoring system needs further evaluation and validation.

In the present review, no studies were identified using standardized forms to assess QoL before and after stent treatment. One randomized study used SF-36 to evaluate the QoL in 10 patients treated with duodenal stents [49], which is a validated and frequently used QoL questionnaire. This study was, however, too small for inclusion in this review. In 16% of the studies, the Karnofsky scale was used, but this scale captures only one aspect of QoL (physical function) and is today considered inadequate for evaluation of QoL [51]. Also for surgical treatment of GOO, data on the effect of QoL is limited [3]. There have been developed and validated several complex and advanced questionnaires for specific symptoms and specific diseases for the assessment of QoL [51]. EORTC C30 and the organ specific modules are now widely used for the evaluation of palliative cancer treatment. By applying these validated tools, the information about the QoL of patients is improved, and a possible discrepancy between the QoL of the patient estimated by the physician and the patient might be revealed. Studies regarding QoL in palliative cancer treatment have shown that physicians tend to overestimate improvement in QoL of the patients [52,53].

Stent-patency related to survival is an important parameter, because the need for re-interventions and re-hospitalizations most likely will reduce the patients QoL. Re-obstruction of the stent by tumor in- and overgrowth is known to occur in 15–20% of the patients [28] and is probably the most important factor influencing stent patency.

The main effect of stent treatment in GOO is re-establishing the passage of food from the stomach to the duodenum. Evaluation of the stent effect can hence be provided by measuring the rate of gastric empting before and after stent placement. None of the reviewed studies included information on this issue. In a recent study by Maetani et al, delayed gastric emptying of a liquid meal after stent placement was demonstrated. The patients resumed oral intake after stenting and those with a severe delay of emptying had a reduced survival time [54]. Rate of gastric emptying was, however, only recorded after stenting, and the quantitative effect of stenting was thus not revealed. More detailed data on the effect of stenting on rate of gastric emptying is thus required, and can be used to improve the knowledge on the relation between GOO and obstructive symptoms. This is an important issue, since the relation between gastrointestinal symptoms and gastric emptying might be rather weak [55]. Furthermore, knowledge concerning the effect of SEMS on gastric emptying could possibly help identifying subgroups of patients, in which stenting is particularly beneficial. Gastric emptying is a complex process involving grinding and emptying of the meal, and it is not likely that the re-establishment of passage is followed by a more rapid rate of gastric emptying in all subjects treated.

## Conclusion

Only 40% of the studies reviewed used a graded scoring system to evaluate the clinical effect of their treatment. Furthermore, most studies using a graded scoring system applied a point score adapted from dysphagia in esophageal cancer and did thereby not address the symptoms more specific for GOO. The presence of obstructive symptoms (severe vomiting, nausea and bloating) is probably severely reducing the patients QoL. In palliative cancer care, improvement of QoL is a main treatment goal, and data on this issue are missing in all the evaluated papers. Objective evaluation of gastric/duodenal function after stenting is limited and no studies have performed quantitative tests of gastric emptying. The present review thus indicates that the available reports do not provide sufficient relevant information of the clinical outcome of duodenal stenting. In future studies, these relevant issues should be addressed to allow improved evaluation of the effect of stent treatment.

## Competing interests

The authors declare that they have no competing interests.

## Authors' contributions

LL performed the systematic search and drafted the manuscript in cooperation with AWM and TH. All three authors have read and approved the final manuscript.

## Pre-publication history

The pre-publication history for this paper can be accessed here:

http://www.biomedcentral.com/1471-230X/9/45/prepub

## Supplementary Material

Additional file 1 **supplementary file including all details concerning the search**. Click here for file
