# Supplementary material for: Treatment of malignant gastric outlet obstruction with stents: An evaluation of the reported variables for clinical outcome
Source: BMC Gastroenterol. 2009 Jun 17;9:45. doi: 10.1186/1471-230X-9-45 (PMC2708180; doi:10.1186/1471-230X-9-45)
Supplement: Additional file 1 — supplementary file including all details concerning the search. [file 1471-230X-9-45-S1.doc]

**Supplementary file including all details concerning the search strategy**

**Search terms**

A search for published literature for the time period January 2000 - September 2007 was conducted in Pub Med, Embase, and Cochrane library combining the following search terms: duodenal stent, malignant duodenal obstruction, gastric outlet obstruction, SEMS, and gastroenteroanastomosis.

Reference lists were hand-searched for additional literature. Furthermore, reference lists of review articles and metaanalyses from the relevant time period were used to identify additional literature. Abstracts were not included.

**Selection/inclusion criteria**: Studies presenting data on clinical outcome/effect after treatment with gastroduodenal stents including ≥ 15 patients and written in English were included in the present review. When studies included identical patients, the most recent study was included.

**Search results**: There were no RCT´s fulfilling the inclusion criteria, so there was no basis for performing a meta-analysis. Both prospective and retrospective studies were included. This paper is a review of the methods in use for reporting the treatments clinical effect.

45 original papers fulfilled the inclusion criteria and were reviewed with regard to the following parameters:

1. The use of a graded scoring system evaluating clinical success

2. Assessment of QoL before and after treatment

3. Information on stent- patency and survival

Stent patency was defined as the time to re-intervention after a successful stent deployment

4. The use of objective criteria to evaluate the stent effect
